# Micro-Opto-Electro-Mechanical Systems for High-Precision Displacement Sensing: A Review

**DOI:** 10.3390/mi15081011

**Published:** 2024-08-06

**Authors:** Chenguang Xin, Yingkun Xu, Zhongyao Zhang, Mengwei Li

**Affiliations:** 1 School of Instrument and Electronics, North University of China, Taiyuan 030051, China; sz202306051@st.nuc.edu.cn (Y.X.); zhangzhongatwork@163.com (Z.Z.); 2 School of Instrument and Intelligent Future Technology, North University of China, Taiyuan 030051, China; 3 State Key Laboratory of Extreme Photonics and Instrumentation, Zhejiang University, Hangzhou 310007, China

**Keywords:** MOEMS, displacement, optical sensing

## Abstract

High-precision displacement sensing has been widely used across both scientific research and industrial applications. The recent interests in developing micro-opto-electro-mechanical systems (MOEMS) have given rise to an excellent platform for miniaturized displacement sensors. Advancement in this field during past years is now yielding integrated high-precision sensors which show great potential in applications ranging from photoacoustic spectroscopy to high-precision positioning and automation. In this review, we briefly summarize different techniques for high-precision displacement sensing based on MOEMS and discuss the challenges for future improvement.

## 1. Introduction

The measurement of displacement is a fundamental activity in various areas of dimensional metrology, which are highly required in both scientific research (e.g., photoacoustic spectroscopy, optical force measurement) and industrial applications (e.g., high-precision positioning, navigation) [1,2,3,4]. In past decades, different technologies have been reported for displacement sensing, which can be generally divided into two categories: optical methods (e.g., laser interferometers, time-of-flight sensors and grating interferometers) and electrical methods (e.g., capacitive sensors, inductive sensors and Eddy current sensors) [5,6,7,8]. Benefiting from properties including non-contact measurement, high resolution, wide bandwidth and long measuring range, the optical approaches have become the preferred methods for ultra-precision displacement measurement in many applications [9]. Despite the great achievement reported in past decades, the optical displacement sensors generally suffer from a relatively larger volume and lower integration compared to the electrical approaches, resulting from a requirement for miniaturized high-performance optical sources and detectors as well as careful alignment between multiple optical components [2,8,9]. Considering the recent interest in developing miniaturized devices and systems with much less power consumption, lower cost and higher functionality, there is a competing need for developing ultra-compact displacement sensors that can provide comparable performances or even unique functions compared to traditional devices [10].

MOEMS have given rise to an excellent platform for miniaturized displacement sensors, which combines the merits of optical measurement and micro-electro-mechanical systems to enable both high performance and compactness [11,12]. In past decades, a wide range of MOEMS sensors has been demonstrated, including displacement sensors, spectrometers, pressure sensors, temperature sensors, accelerometers and gyroscopes [13,14,15,16,17,18,19,20]. In past decades, MOEMS displacement sensors based on different principles, such as optical interference, optical reflection, grating diffraction, evanescent coupling and bandgap modulation, have been reported [21,22,23,24,25]. The advantages of these MOEMS displacement sensors involve their small volume, low weight, low power consumption, high resolution and high repeatability in fabrication, making these devices highly promising in applications such as defense, consumer electronics and medical equipment [26]. However, a systematical analysis of the reported MOEMS displacement sensing techniques has not been found yet.

**Figure 1:**
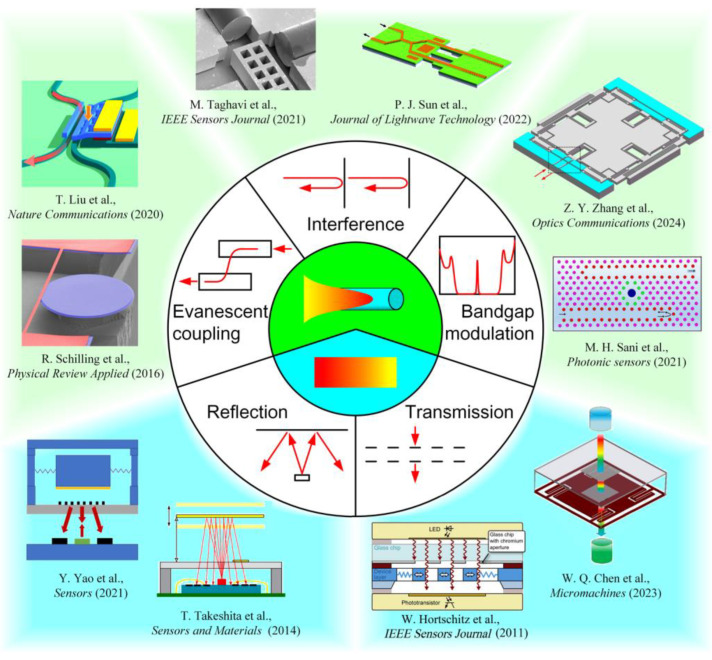
Classification of MOEMS displacement sensing techniques [25,27,28,29,30,31,32,33,34,35].

This paper aims to address the pressing issue of the current lack of systematic review for MOEMS displacement sensing. According to different optical measurement principles, we divide MOEMS displacement sensing techniques into two categories: the guided-wave-based and the free-space-based (as shown in Figure 1). The guided-wave-based sensors include the evanescent coupling scheme, the interference scheme and the bandgap modulation scheme. And the guided-wave-based types can be further categorized into the reflection scheme and the transmission scheme. The principles, structures, performances, advantages and disadvantages of state-of-the-art MOEMS displacement sensing techniques are discussed. This paper also presents the development tendency to meet the ever-increasing demand for integrated high-precision displacement measurement.

## 2. Definition of MOEMS Displacement Sensing

Since displacement measurement is the basis for the measurement of different physical quantities such as distance, surface profile, velocity, vibration, accelerated velocity and rotation, many other MOEMS sensors (e.g., pressure sensors, accelerometers and gyroscopes) are also operated based on measurement of displacement [5,11,36]. For example, the angular rate can be calculated from the linear displacement of a proof mass in a Coriolis-effect-based MOEMS gyroscope [36]. In this review, we mainly focus on MOEMS displacement sensors. Other relative MOEMS devices, such as accelerometers and gyroscopes, may also be mentioned to help understand the principles, structures, performances and applications of MOEMS displacement sensing techniques. However, only the parameters related to displacement sensing are presented and discussed. The devices mentioned below are typically fabricated by standard CMOS processes, which are beyond the scope of this review. The authors refer readers to the works of H. Qu (2016) and J. Zhu et al. (2020) [26,37]. These papers contain the expressions for the fabrication of relative devices.

## 3. Performance Indexes

**Figure 2:**
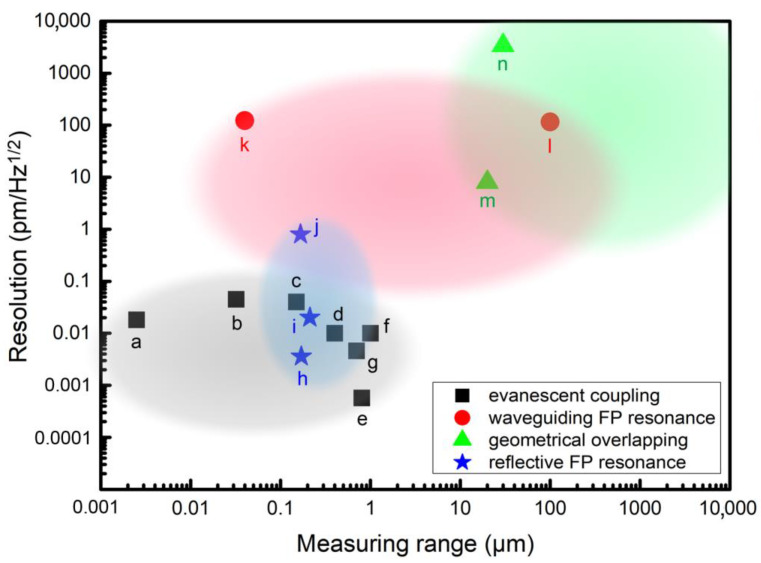
Displacement resolution with corresponding measuring range for MOEMS displacement sensing techniques based on different principles, including evanescent coupling, waveguiding Fabry-Perot (FP) resonance, geometrical overlapping and reflective FP resonance. (a-[41], b-[27], c-[43], d-[44], e-[42], f-[28], g-[23], h-[24], i-[45], j-[46], k-[47], l-[48], m-[29], n-[49]).

Technical specifications, including resolution, sensitivity, accuracy and measuring range, are the most important performance indexes of displacement sensing [38]. The resolution is defined as the smallest change in the displacement being measured that causes a perceptible change in the corresponding output (e.g., voltage). It corresponds to the limitation of detecting. Typically, the resolution depends on the internal noises in a MOEMS sensor, including electronic, mechanical and optical noises [39]. Since the output of most MOEMS displacement sensors is electrical, it is common practice to express the noises in m/Hz^1/2^ [40]. In past decades, resolution down to 0.1–10 fm/Hz^1/2^ level has been reported in MOEMS sensors (as shown in Figure 2) [41,42]. The sensitivity is the linear correlation coefficient between the output signal and the input displacement, which is typically expressed in units such as V/nm, nm/nm or %/nm. A larger sensitivity generally leads to a higher signal-to-noise ratio, related to a higher resolution [36]. The accuracy is defined as the closeness of agreement between a measured quantity value and a true quantity value for the measured displacement, which is typically quantified by measuring error. The measuring range refers to the variation between the lower and the upper limits of indication, generally limited by geometries such as the length of the optical path. Considering the ultra-compact structure and small size of MOEMS sensors, the measuring range of these sensors typically ranges from sub-micrometer to millimeter level.

It is worth mentioning that, for displacement measurement, the performance indexes mentioned above can hardly be treated independently [5]. For example, the noise not only determines the value of resolution but also influences the accuracy. And the relative accuracy is highly related to measuring range. For example, the same accuracy of 1 nm for two different measuring ranges of 1 μm and 1 mm refers to a different relative error of 0.1% and 0.0001%, differing in three orders of magnitude. As a result, the accuracy and resolution are recommended to be compared within a comparable measuring range for different MOEMS displacement sensors [9].

## 4. Classifications

The MOEMS displacement sensing technologies mentioned in this review are categorized into a guided-wave-based scheme and a free-space-based scheme according to different working principles. The guided-wave-based techniques can further be categorized into different schemes, including the evanescent coupling scheme, the interference scheme and the bandgap modulation scheme. Similarly, the free-space-based techniques can also be categorized into the reflection scheme and the transmission scheme. Both the measuring principles, structures and performances for different schemes are discussed in this chapter.

### 4.1. Guided-Wave Based Techniques

The guided-wave-based MOEMS displacement sensing techniques are generally based on optical waveguides such as slab waveguides, optical fibers and photonic crystal waveguides. Using optical coupling between different waveguides or optical resonance in microcavities, displacement can be measured by detecting the change of optical transmission or reflection caused by the change of geometries, such as coupling length and cavity length. A high resolution (e.g., going down to 0.1–10 fm/Hz^1/2^ level) within a small measuring range, typically less than 1 mm, has been demonstrated [41,42]. In these cases, high-precision control of waveguiding properties such as waveguiding modes and resonant frequencies is generally required.

#### 4.1.1. Evanescent Coupling Scheme

As the size of optical waveguides goes down to be comparable to or less than the wavelength of guided light, a large amount of the light will propagate outside the waveguides as evanescent waves, which makes nano-/microwave-guides being excellent platform for optical sensing [50,51]. A tunable coupling efficiency between different nano-/microwave-guides has been demonstrated by changing overlapping lengths, indicating the possibility for displacement measurement by detecting the optical transmission or reflection of an optical coupler consisting of nano-/microwave-guides [52]. The displacement sensing techniques based on evanescent coupling can be achieved by using either a non-resonant structure or a resonant structure. In the non-resonant cases, multiple parallel nano-/microwave-guides are generally used, showing a relatively simple structure with a large optical bandwidth [27]. Both the relative displacement between the waveguides in the axial or the lateral direction can be measured by detecting the optical transmission or reflection. For the resonant scheme, microcavities are typically used. Pumping light is coupled into/out an optical cavity by using a nano-/microwave-guide [53]. With a relative displacement between the cavity and the waveguide, the optical transmission/reflection changes. Despite the high sensitivity, the resonant nature of the cavity response intrinsically limits the dynamic range and optical bandwidth [27,54].

**Figure 3:**
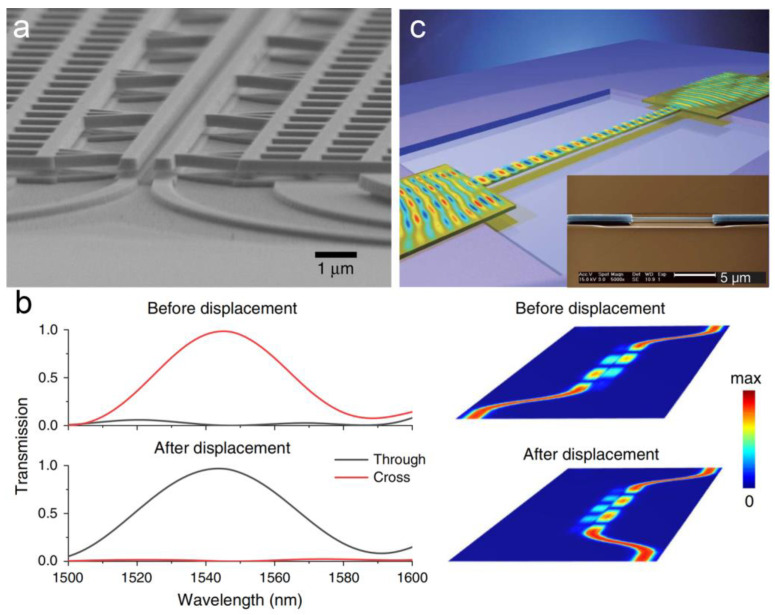
(**a**) Scanning electron microscope (SEM) image of a nanomechanical directional coupler consisting of two nano-waveguides [27]. (**b**) Simulated transmission and electric field distribution before and after a displacement of 55 nm for the coupler shown in (**a**) [27]. (**c**) Three-dimensional schematic illustration of a substrate-coupled free-standing waveguide [41]. Inset is the SEM image correspondingly.

The evanescent coupling process between different optical nano-/microwave-guides has been studied in both theory and experiment, showing an optical sensitivity for the change in coupling efficiency to overlapping length at 10%/μm level with a maximum coupling efficiency over 95% [52,55,56,57]. In 2020, T. Liu et al. demonstrated an integrated MOEMS displacement sensor (as shown in Figure 3a) [27]. By using a three-dimensional directional coupler fabricated on an InP/InGaAsP multi-layer stack grown by metalorganic vapor-phase epitaxy, the sensor showed a displacement imprecision down to 45 fm/Hz^1/2^ within a measuring range of ~32 nm. Benefiting from the non-resonant operating, an ultra-wide optical bandwidth of ~80 nm was obtained, which can greatly release the high requirements on high-performance read-out systems (as shown in Figure 3b). Later, C. Xin et al. designed a MOEMS accelerometer based on displacement measurement using evanescent coupling through silicon nano-waveguides in the year 2022 [58]. A sensitivity of 32.8%/μm was obtained in the simulation. Photonic crystal waveguides have also been used. In 2007, the relationship between the input displacement to the output of a photonic crystal co-directional coupler was discussed, indicating the possibility of photonic crystals in displacement sensing [59]. In 2012, A.G. Krause et al. reported a MOEMS accelerometer making use of ultrasensitive displacement read-out with noise imprecision down to 4 fm/Hz^1/2^ based on a photonic crystal nanocavity [60]. In 2019, E. Soltanian et al. demonstrated a differential MOEMS accelerometer based on the displacement-induced transmission change of an optical coupler formed by a two-dimensional photonic crystal structure [61]. An optical sensitivity of ~3.3%/nm within a range of ±15 nm was demonstrated. An end-to-end structure has also been used to measure tiny displacement. In 2009, M. Li et al. reported the measurement of relative displacement between nanocantilevers [43]. Two nanocantilevers, which were also used as two optical waveguides, were located end-to-end with a 200 nm gap between them. With an out-of-plane displacement on one of the nanocatilevers, the transmission from one cantilever to another changed, resulting in a measurement with a sensitivity of 40 fm/Hz^1/2^.

By using evanescent coupling to a substrate, the displacement measurement can also be operated by using only one single nano-waveguide, leading to a simpler structure and higher compactness [62,63]. In 2008, M. Li et al. reported their remarkable work on the detection and exploitation of transverse optical forces in an integrated silicon photonic circuit through the displacement measurement of a nano-waveguide (as shown in Figure 3c) [41]. The device was fabricated by standard electron beam lithography and dry etching processes on silicon-on-insulator wafers. By using evanescent coupling between a single mode strip nano-waveguide to a dielectric substrate, transmission amplitude changed as an optical force arising from the waveguide. In this way, only one waveguide was needed. A displacement sensitivity of 18 fm/Hz^1/2^ was obtained experimentally with a tiny displacement of ~2.5 nm.

**Figure 4:**
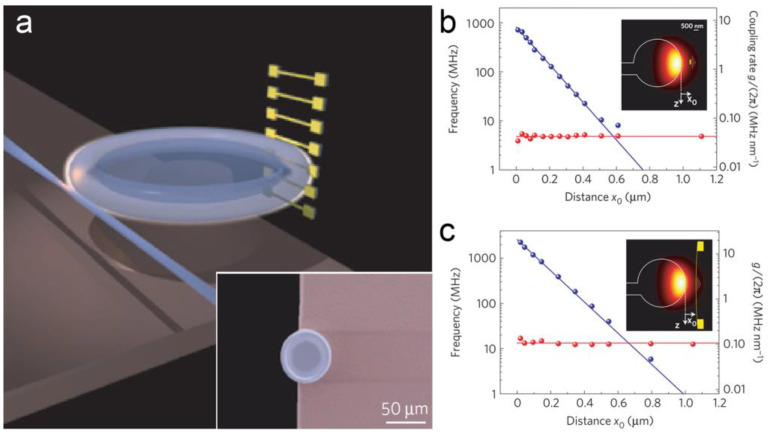
Displacement sensing based on a near-field coupled optical cavity [42]. (**a**) Schematic diagram of an optical cavity coupled with a nano-waveguide. Inset is the SEM image of the cavity. The experimental relationship of the linewidth (red) and the negative optical frequency shift (blue) of the cavity on x_0_, which is the distance between the optical cavity to (**b**) a Si_3_N_4_ string and (**c**) a sheet, respectively.

By using resonant enhancement (as shown in Figure 4), resolution can be significantly improved [42,64,65]. In 2009, G. Anetsberger et al. demonstrated a near-field cavity optomechanical system formed by a silica microcavity, in which a resolution at sub-fm/Hz^1/2^ level was reported [42]. In 2012, a micro-electro-mechanically controlled cavity-based optomechanical sensing system was demonstrated by H. Miao et al., reporting a displacement resolution of 4.6 fm/Hz^1/2^ [23]. In 2016, R. Schilling et al. proposed a monolithic integration of a high SiN resonator and a SiO_2_ microdisk cavity by using a chemical-mechanical-polishing process [28]. Without deteriorating the intrinsic Q of the resonator and the cavity, a displacement noise down to 10 fm/Hz^1/2^ was demonstrated. MOEMS accelerometers based on the displacement measurement by using ring resonators have also been designed and simulated. The change in acceleration can be read out by detecting the relative displacement of the proof mass as a shift in the resonant wavelength [66]. In 2022, R. Gholamzadeh et al. designed a light-intensity modulated MOMS accelerometer [67]. The effective refractive index of the resonator changed with an acceleration-induced displacement of the proof mass, leading to a wavelength shift in spectra. An optical sensitivity of 0.7%/nm was obtained in the simulation. In 2023, Y. Feng et al. reported a MOEMS accelerometer by mode localization for high-sensitivity measurement using cascaded ring resonators [68]. The acceleration was measured from the change in coupling length caused by the inertial displacement through a spring–mass system.

**Figure 5:**
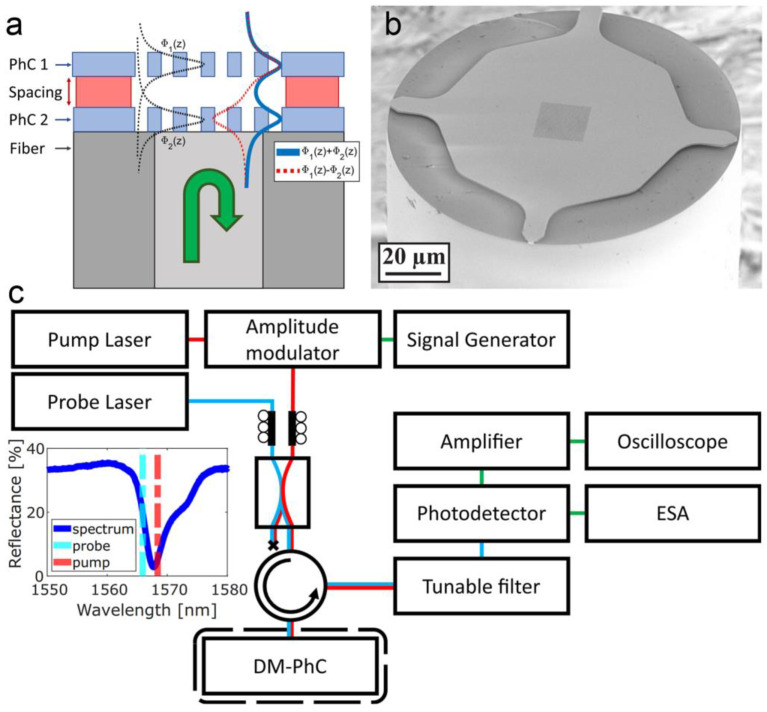
A fiber-tip MOEMS sensor based on evanescent coupling between two overlapping photonic crystal modes [44]. (**a**) Design of the sensor. (**b**) SEM image of the sensor. (**c**) Setup used for the driven measurements.

It is worth mentioning that an ultracompact MOEMS sensor has been demonstrated by placing a double-layer structure on the tip of an optical fiber (as shown in Figure 5) [44]. An InP double-layer photonic crystal structure was fabricated on the tip of a single-mode fiber. The evanescent fields of the two photonic crystal modes overlapped with each other and formed a coupled system in which the resonant frequencies of the modes were affected by the separation between two photonic crystal layers. With no need for coupling optics, this device shows high functionality, ultra-small footprint, low-cost read-out as well as a displacement imprecision down to 10 fm/Hz^1/2^.

#### 4.1.2. Interference Scheme

For the interference scheme, incoming light is generally divided into two parts by a beam splitter. The relative displacement causes a change in path length or refractive index, leading to an additional difference in phases between the two optical paths. This change in the phase can be read out by an optical interference process. Different interference structures such as FP interferometer, Michelson interferometer and Mach–Zehnder interferometer have been demonstrated for MOEMS displacement sensing.

**Figure 6:**
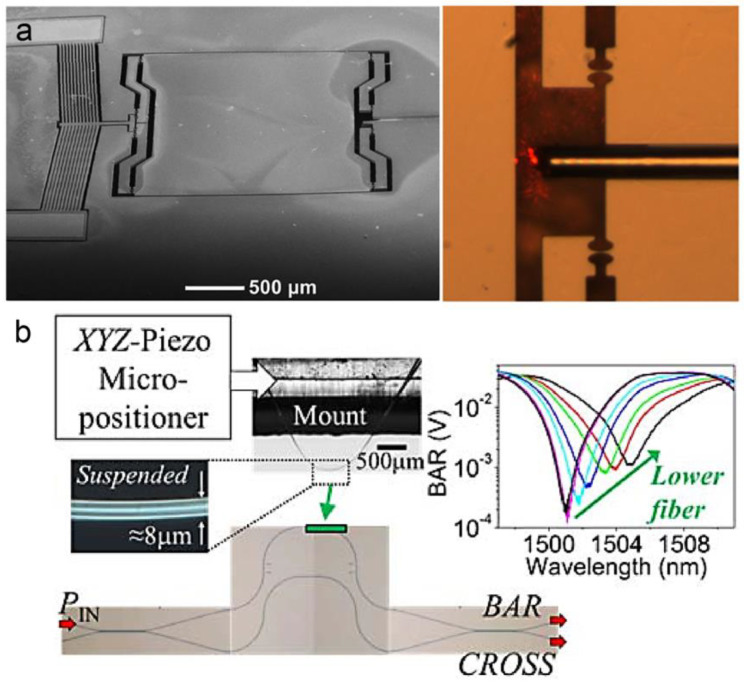
(**a**) The SEM image of a MOEMS FP interferometer. Inside is the optical image of an assembled optical fiber [73]. (**b**) Optical image of a MOEMS Mach–Zehnder interferometer [47].

For FP interferometers, a cavity is generally fabricated by using two reflectors consisting of the sidewall of a mass proof and the end of a cleaved optical fiber. The optical fiber is hybrid integrated into on-chip structures. The resonance wavelength (*λ_res_*) shifts with a change in the cavity length (Δ*L*), which can be given by [69]

(1) $$\Delta \Phi =\frac{4\pi n\Delta L}{\lambda_{res}}$$

where *n* is the refractive index of the cavity medium, Δ*Φ* is the phase difference. As the distance between the cleaved optical fiber and the sidewall changing, the power coupled back into the fiber changes as well. As a result, displacement can be measured in an amplitude-modulated process [70,71]. Benefiting from the FP resonance, a high resolution can be obtained [48,72]. In 2018, Y. Kim et al. proposed an optical fiber FP micro-displacement sensor for MEMS in-plane motion stage [73]. By using polished optical fiber and the sidewall to form an optical cavity, both the magnitude and the direction of the displacement can be obtained from interferometric signals. An accuracy below 35 nm within a range larger than 3 μm was obtained. The optical fiber was assembled inside a trench to be integrated with on-chip structures (as shown in Figure 6a). Similar principles have also been used in MOEMS accelerometers and gyroscopes [36,74,75]. For example, in 2016, O. Kilic et al. reported a MOEMS vibratory gyroscope based on two hybrid FP cavities consisting of gold-coated optical fibers and a reflective surface on a substrate [54]. An equivalent displacement of Brownian noise was calculated to be 1.4 × 10^−4^ nm/Hz^1/2^. In 2021, a dual-axis MOEMS accelerometer was demonstrated experimentally by M. Taghavi et al. [30]. By using two cleaved optical fibers located perpendicularly, two freedom-of-degree measurements were demonstrated within a range of ~190 nm. In the next year, M. Rahimi et al. reported a differential MOEMS accelerometer based on two FP micro-cavities formed between the proof mass and the optical fiber end faces [76]. Two optical fibers were located on opposite sides of the device, leading to a differential output. Consequently, the measured spectral shift doubled the sensitivity compared to the single-cavity cases. An optical sensitivity of ~0.4%/nm within a range of ±130 nm was demonstrated.

For Michelson and Mach–Zehnder interferometers, the waveguides need to be carefully designed to obtain a maximum difference in phase between two optical paths. In these cases, Δ*Φ* can be given by [74]

(2) $$\Delta \Phi =\frac{2\pi }{\lambda_{0}}n_{eff}\Delta D$$

where $n_{eff}$ is the effective refractive index of the optical waveguides, ΔD is the change of difference in optical paths, $\lambda_{0}$ is the wavelength of the input light. In 2022, P. Sun et al. obtained an optical displacement sensitivity of 13.7%/nm in a MOEMS accelerometer by using an on-chip Michelson interferometer [25]. In 2014, M.W. Pruessner et al. reported an unbalanced Mach-Zehnder interferometer for chip-scale displacement sensing (as shown in Figure 6b) [47]. Associated with a substrate-coupling design, a displacement sensitivity of 0.75 mV/nm within a range of 40 nm was obtained. And the displacement noise was 123 pm/Hz^1/2^.

#### 4.1.3. Bandgap Modulation Scheme

Recently, displacement sensing techniques based on photonic crystals have been developed based on different principles, including evanescent coupling, resonant enhancement and bandgap modulation [22,60,77,78]. Among the techniques mentioned above, the approaches based on bandgap modulation have shown high compactness with a simple waveguiding structure. Typically, the displacement measurement is operated by wavelength modulation of a photonic bandgap with an optical sensitivity ranging from 0.1 to 1 nm/nm level. The photonic bandgap is defined as a range of wavelengths that cannot be propagated in a photonic crystal. Arising from the destructive interference occurring between the emitting and reflective waves on the boundaries where the refractive index changes, it can be greatly influenced by the geometries of photonic crystals [79,80]. Introducing certain defects by changing the materials or the geometries of photonic crystals, transmitted defect modes can be observed within the photonic bandgap by breaking the periodicity of the structure [31]. The wavelength of these modes is related to the displacement-induced changes (e.g., cavity length and thickness of air gap) in the photonic crystal [81]. Generally, MOEMS displacement sensing techniques based on bandgap modulation can be divided into two categories by different structures, such as quasi-FP cavities and finger-associated filters.

**Figure 7:**
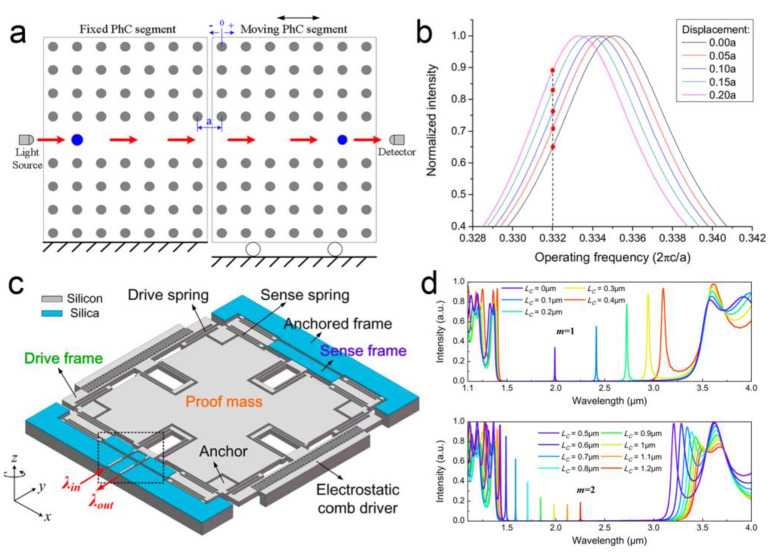
(**a**) Schematic diagram of a line-defect photonic crystal cavity [83]. (**b**) Simulated relationship between the normalized intensity to the operating frequency with different input displacements in the line-defect cavity [83]. (**c**) Schematic diagram of a MOEMS gyroscope based on photonic crystal resonant cavity formed by two distributed Bragg reflectors [32]. (**d**) Simulated output spectra of the MOEMS gyroscope [32].

For the quasi-FP cavities, a double-layer photonic crystal structure is generally used. Although somewhat similar to the traditional FP cavities, this structure shows some differences: (1) less perpendicular leakage; (2) different transmission properties; (3) a much smaller operating distance, indicating a high sensitivity without long operating distance and high reflectivity [82,83]. Quasi-FP cavities were first reported for displacement sensing based on a free-space structure by using evanescent tunneling between two photonic crystal slabs [84]. After that, waveguiding quasi-FP schemes were proposed for higher sensitivity and compactness. Both two-dimensional (2D) lattice arrays and one-dimensional (1D) waveguides have been reported. For the lattice-array-based cases, the waveguides were fabricated by moving certain lattices in a 2D array. For example, using a line-defect resonant cavity consisting of two co-planar photonic crystal waveguides, in-plane displacement sensing has been demonstrated (as shown in Figure 7a,b) [83,85]. A quasi-linear relationship of the relative displacement (Δd) between the two waveguides on the transmission was found within a range of up to a few micrometers. The change of the transmission (ΔT) can be given by [83]

(3) $$\Delta T=\frac{3\sqrt{3}QM}{4\omega_{0}}\Delta d$$

(4) $$M=\frac{\Delta \omega }{\Delta d}$$

where Q is the quality factor, $\omega_{0}$ is the resonant frequency, Δω is the shift of the resonant frequency. A simulated sensitivity of ~1.15/*a*, where *a* is the lattice constant, was obtained.

Compared to the lattice-array-based approaches, most of the works focused on 1D photonic crystal waveguides. In these cases, only a few periodical lattices are required to be fabricated inside a waveguide, resulting in a much higher compactness and functionality. The wavelength of the left ($\lambda_{l}$) and the right ($\lambda_{r}$) band edge of the photonic bandgap can be given by [80]

(5) $$\lambda_{l}=\left(\pi n_{H}d_{H}+n_{L}d_{L}\right)/\cos^{-1}\left(-\rho \right)$$

(6) $$\lambda_{r}=\left(\pi n_{H}d_{H}+n_{L}d_{L}\right)/\cos^{-1}\left(\rho \right)$$

where $n_{H}$ and $n_{L}$ is the refractive index of high-index and low-index materials used in the waveguide, respectively, $d_{H}$ and $d_{L}$ is the thickness of one single cell fabricated by the two materials respectively, and ρ = ($n_{H}-n_{L}$)/($n_{H}$ + $n_{L}$). In 2019, K. Huang et al. proposed a MOEMS accelerometer with one single-proof mass [86]. Simulated results show an optical sensitivity of 1.23 nm/nm within a range over 1 μm. In the next year, the same group improved the optical sensitivity to 1.41 nm/nm [87]. A broad optical bandwidth from 1.2 μm to 2.7 μm was also demonstrated in the simulation. In 2012, K. Zandi et al. reported a MOEMS accelerometer based on a photonic crystal resonant cavity formed by two distributed Bragg reflectors [77]. In 2024, Z. Zhang et al. designed a MOEMS gyroscope using a similar structure (as shown in Figure 7c,d) [32]. A simulated optical sensitivity of 1.2 nm/nm was obtained.

**Figure 8:**
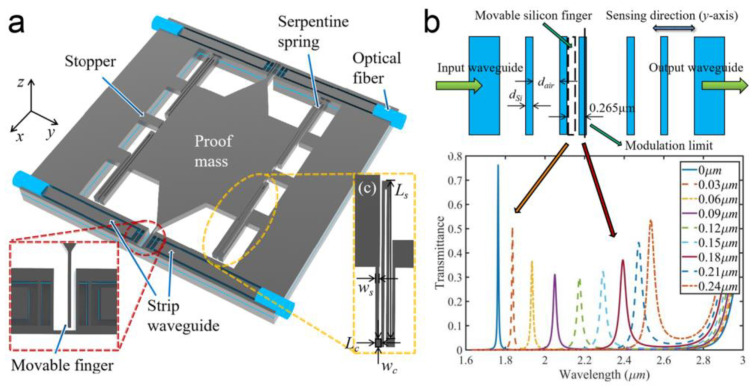
A MOEMS accelerometer based on a finger-associated filter [92]. (**a**) Schematic diagram of the accelerometer. (**b**) Simulated transmission with different displacements of the finger.

For the finger-associated filters, a movable finger is usually located inside a photonic crystal cavity. As a result, optical defect modes appear within the transmitted spectra. The wavelength of these modes is modulated by the displacement of a finger rather than the length of the cavity in this case [88]. The influence of a single point defect inside a photonic crystal waveguide between two reflectors on resonant wavelengths has been investigated in theory [89]. In 2016, A. Sheikahaleh et al. designed a MOEMS accelerometer in which a silicon finger attached to a proof mass was located inside a 1D photonic crystal waveguide [88]. The guided light passing through the waveguide was detected. The periodicity of the photonic crystal was broken by the movable finger, leading to a defect mode within the photonic bandgap in the transmission spectra. Later, they improved the device by using a multi-layer graphene finger [90]. With a lateral displacement of the graphene finger, the overlap between the finger and the photonic crystal changed. Using the optical absorption of the graphene, the transmission of the photonic crystal decreased. An optical sensitivity of ~1.5%/μm was obtained theoretically. In the next year, in-plane displacement measurement based on a similar graphene-based design was also reported by a wavelength modulation process, showing an optical sensitivity of ~0.46 nm/nm [91]. In 2021, K. Huang et al. improved the optical sensitivity to 4.42 nm/nm in a double-mode MOEMS accelerometer by using double fingers (as shown in Figure 8) [92,93]. As the width of the silicon/air layer was determined by a quarter of the primary wavelength of the coupled light, the simulated photonic bandgap reached a high value across wavelength from 1.1 μm to 2.75 μm. In 2024, X. Yang et al. proposed a MOEMS gyroscope with a simulated optical sensitivity of 1.11 nm/nm within a measuring range of ±350 nm [20]. Other structures, such as WGM cavities, have also been used for displacement sensing by introducing a movable defect inside the cavities [31,94]. However, the optical bandwidth and optical sensitivity (e.g., at 0.01–0.1 nm/nm level) is generally lower than that in the quasi-FP case.

### 4.2. Figures, Tables and Schemes

MOEMS displacement sensors based on a free-space structure generally consist of a displacement-sensing chip, which translates displacement to optical signals, and a reading head formed by an optical source and detector. The displacement-sensing chips are usually fabricated from plane components such as gratings, mirrors and photonic crystal slabs. The displacement induces a change of geometries such as air gap, in-plane distance and overlapping area, leading to a different intensity of reflected, diffracted or transmitted light. A relatively large measuring range of up to several millimeters has been reported [95]. Considering an intensity modulation used in most cases, light-emitting diodes or vertical-cavity surface-emitting lasers are widely used. Divided by the location of the reading head, these sensors can be classified into two categories: the transmission scheme and the reflection scheme.

#### 4.2.1. Transmission Scheme

For the transmission scheme, the optical source and detector are generally located on opposite sides of the displacement-sensing chip. The exposing light from the optical source is detected after passing through the sensing chip. Benefiting from a structure based on a coaxial optical path, the transmission scheme usually shows a simple multi-layer structure. The displacement of the proof mass changes the optical transmission based on different principles, such as geometrical overlapping and the Talbot effect.

**Figure 9:**
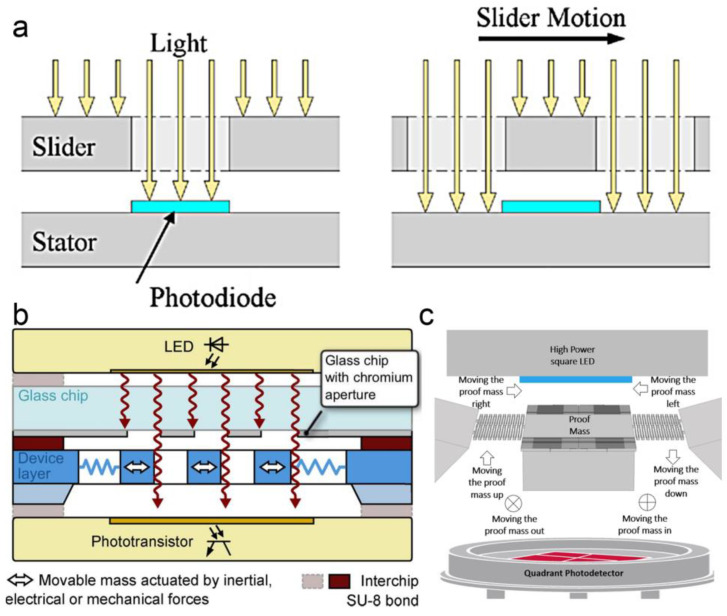
(**a**) Measuring principle of geometrical-overlapping based MOEMS displacement sensing [95]. (**b**) Schematic setup of a MOEMS vibration sensor based on 2D rectangle arrays [29]. (**c**) Schematic setup of a MOEMS accelerometer based on optical blocking of a proof mass [96].

For displacement sensing based on geometrical overlapping, the input displacement changes the overlapping area between different components (as shown in Figure 9a). With a different overlapping, the different fraction of light is blocked, resulting in a change of optical transmission. A linear relationship between the transmission and the displacement is generally presented as [29]

(7) ΔU∝ΔA=L·Δd

where ΔU is the output of the photodetector, ΔA is the change in the overlapping area, and *L* is the length of the optical aperture. For example, in 2009, M. Beyaz et al. reported a miniaturized positioner using integrated photodiode sensors [95]. The light passed through an array of through holes located on a movable substrate. After that, it was detected by a photodiode array. As the substrate slid linearly, the alignment between the through holes and the photodiode array changed, resulting in a different output from the photodiodes. A relatively large measuring range of up to 4 mm was obtained with a resolution at the micrometer level. In 2011, W. Hortschitz et al. reported a MOEMS sensor for relative displacement measuring (as shown in Figure 9b) [29]. Two-dimensional rectangle arrays with a period of 40 μm were fabricated on two substrates, respectively. The relative displacement between the two substrates resulted in a linear change of overlapping area. A noise of 8 pm/Hz^1/2^ was obtained. Later, they improved the device by using a triangle array [49]. A non-linear transfer characteristic was achieved by pairing the triangle array with a rectangular array. Both the static displacement and the vibration of the proof mass were obtained by using different harmonics of the output signals. A resolution down to 3.67 nm with a noise of 3.41 nm/Hz^1/2^ was demonstrated. In 2022, S. Abozyd et al. designed a MOEMS accelerometer for three freedom-of-degree measurements based on optical blocking of a proof mass (as shown in Figure 9c) [96]. The proof mass was held between a light-emitting diode and a quadrant photodetector. By analyzing the output intensities of different detectors, displacement measurement with a range of up to 3 mm was achieved.

**Figure 10:**
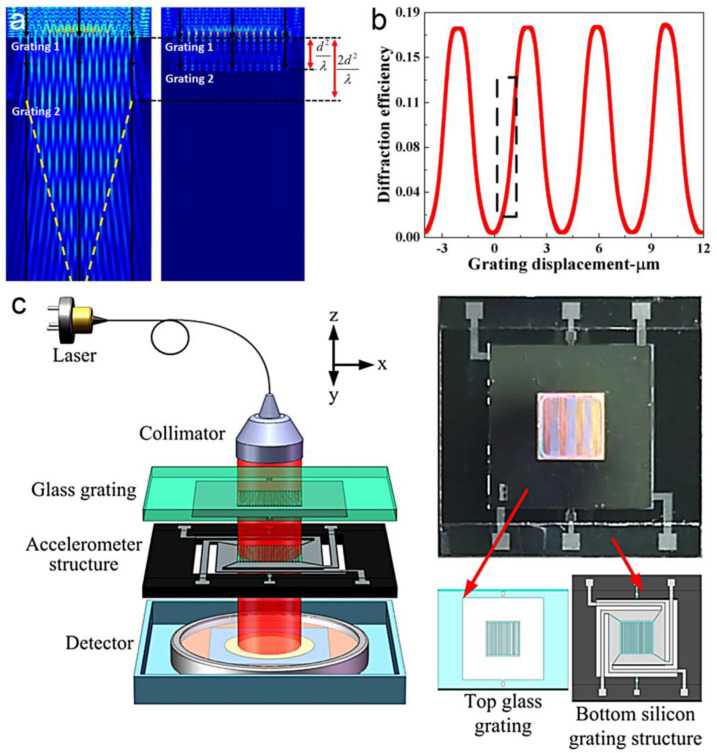
(**a**) Measuring principle of Talbot-effect-based MOEMS displacement sensing [103]. (**b**) Simulated optical transmission of double-layer gratings with a relative displacement [33]. (**c**) Schematic setup of a MOEMS accelerometer based on Talbot effect of optical gratings [103].

Optical gratings have been widely used in displacement sensing. Optical interferometers and encoders based on optical gratings have been reported in past decades [38,97]. In recent years, displacement sensing based on the Talbot effect of micro-gratings has attracted continuous attention. Optical gratings with a period comparable to the wavelength of the exposing light are preferred for a higher resolution [98]. A periodical optical field behind the first grating is modulated by the second grating with the same period as the first one (as shown in Figure 10a) [99]. The complex amplitude distribution behind the second grating can be defined as [100]

(8) $$U\left(x,z,\Delta d\right)=\exp\left(\mathrm{ik}z\right)\sum_{n=-\infty }^{\infty }\sum_{m=-\infty }^{\infty }C_{n}C_{m}\exp\left[i2\pi \frac{\left(\frac{n}{\cos\theta }\right)+m}{\Lambda }x\right]\exp\left[i2\pi \frac{\frac{n}{\cos\theta }}{\Lambda }\Delta d\right]$$

where θ is the relative rotating angle between the two gratings, Λ is the period of the gratings. As a result, a sinusoidal signal can be obtained from a photodetector as the gratings move relatively to each other in the in-plane direction perpendicular to the grating lines (as shown in Figure 10b). Generally, an interpolation circuit is used to translate the sinusoidal signal into square signals. The displacement can be calculated by counting the square signals. The resolution (S) is given by [97]

(9) $$S=\frac{\Lambda }{C}$$

where C is the subdividing factor of the interpolation circuit. With a smaller grating period, there is a smaller resolution as well. By using a simple coaxial, optical structure consisting of a laser source, double-layer gratings and a quadrant detector, this method shows an ultracompact structure and high stability compared to traditional diffractive interference optical grating encoders [101]. In 2018, M. Li et al. discussed displacement measurement based on a double-grating model, showing a simulated sensitivity of 0.29%/nm and 1.63%/nm for in-plane and out-of-plane displacement, respectively [102]. Later, the same group reported a single-axial MOEMS accelerometer based on the Talbot effect of 4 μm-period gratings with a sensitivity over 0.02%/nm (as shown in Figure 10c) [33,103]. The double-layer gratings were fabricated by a photolithography process. In 2024, L. Jin et al. demonstrated a tri-axial MOEMS accelerometer [104]. Using three groups of grating pairs located on two substrates respectively, this device can be used to measure the acceleration in three different directions synchronously. A simulated sensitivity of 0.036%/nm and 0.006%/nm for in-plane and out-of-plane directions were obtained, respectively.

#### 4.2.2. Reflection Scheme

Despite a simple coaxial structure, the transmission scheme suffers from a configurational nature in that the components (e.g., optical source and detector) of the reading head have to be located at opposite sides of a displacement sensing chip. It brings in deleterious consequences in some applications because the displacement can only be input from the sidewall [105]. By using a reflective structure, a more compact reading head can be achieved for higher functionality [106]. Microgratings, membranes and metal films are usually used as reflectors. Based on different principles, including FP resonance, diffracted interference, Wood’s anomalies, triangulation measuring and evanescent tunneling, the MOEMS displacement sensing using reflective structures has been demonstrated.

**Figure 11:**
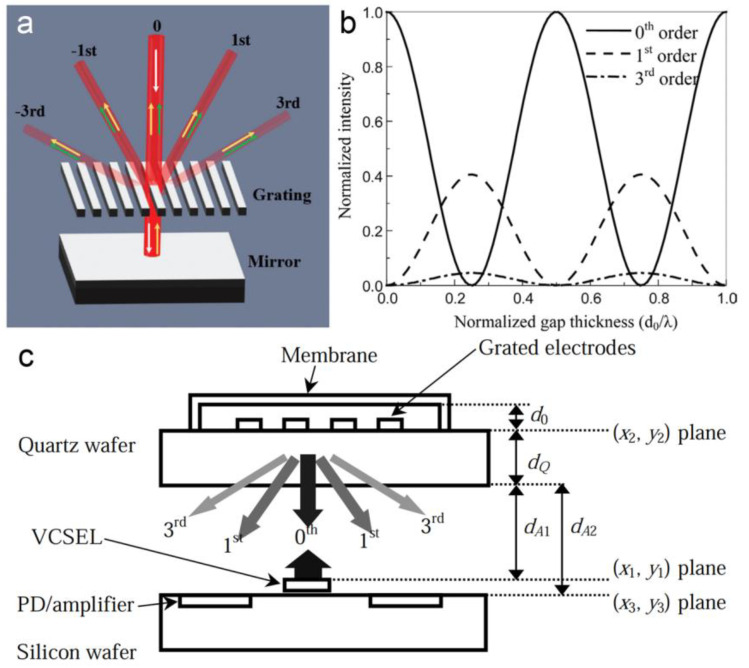
(**a**) Principle of displacement sensing based on asymmetric FP resonance [115]. (**b**) The relationships between the intensity of different diffracted orders to the gap thickness between two reflectors [45]. (**c**) Schematic diagram of a MOEMS displacement sensor based on asymmetric FP resonance [45].

**Figure 12:**
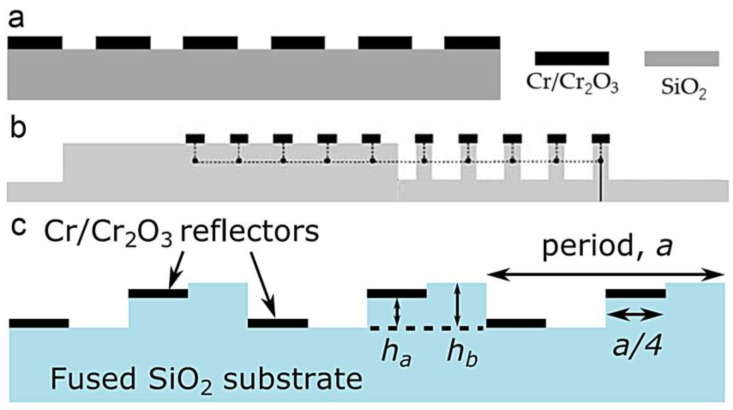
Different optical gratings are used in the displacement sensing based on asymmetric FP resonance. (**a**) Traditional regular grating [34]. (**b**) Quadrature phase-shift dual grating is used to generate quadrature outputs [21]. (**c**) Four-region diffraction grating is used to eliminate the 0th-order diffracted beam [24].

Using an asymmetric FP cavity formed by optical gratings or reflective films, the distance between the two reflectors can be measured by detecting the optical reflection. As an optical grating is exposed to a coherent light, a fraction of light passes through the grating while the other is reflected. And the reflected light will be divided into several diffracted orders. The light passing through the grating is reflected by another reflector, leading to an additional phase. As the distance between the grating and the reflector changes, the optical interference between the reflected lights causes a change in the intensity of the diffracted beams (as shown in Figure 11a,b) [107]. The intensity of the zero ($I_{0}$) and the first ($I_{1}$) diffracted order can be given by [45]

(10) $$I_{0}=I_{in}cos^{2}\left(\frac{2\pi d_{0}}{\lambda_{0}}\right)$$

(11) $$I_{1}=\frac{I_{in}}{\pi^{2}}sin^{2}\left(\frac{2\pi d_{0}}{\lambda_{0}}\right)$$

where $I_{in}$ is the intensity of the input light, *d*_0_ is the distance between the grating and the reflector, $\lambda_{0}$ is the wavelength of the input light. For example, in 2002, C. Savran et al. reported a differential MOMES sensor for nanoscale displacement measurement [46]. By using two adjacent optical gratings with a period of 10 μm, a resolution defined by a noise of 0.8 pm/Hz^1/2^ was demonstrated. In the next year, N.A. Hall et al. reported a displacement resolution down to 0.02 pm/Hz^1/2^ using an FP cavity consisting of a conductive membrane and a reflective optical diffraction grating with a period of 4 μm (as shown in Figure 11c) [45]. Later, the same group improved the device by using a quadrature phase-shift dual grating to replace the traditional regular grating (as shown in Figure 12a,b) [21]. Benefiting from the quadrature outputs, the detecting range was expanded to be over $\lambda_{0}$/4. In 2009, B. Bicen et al. successfully applied this method for a directional optical microphone with a dynamic range of up to 10^4^ Hz [108]. In 2017, R.P. Williams et al. reduced the 0th-order diffracted component by imparting a half-wavelength phase shift to a portion of the reflected light (as shown in Figure 12c) [24]. In this way, an improved resolution of 3.6 fm/Hz^1/2^ was obtained. MOEMS accelerometers and gyroscopes based on the same principle have also been reported [109,110,111,112]. By using an improved mechanical structure such as a gram-scale proof mass and a redesigned spring structure, the MOEMS accelerometers with higher sensitivity are in highly expected [34,113]. In 2024, G. Li et al. designed a wavelength-modulated MOEMS accelerometer by using a covering glass rather than a grating as an optical splitter [114]. By measuring the transmitted spectra, an optical sensitivity of 1.98 nm/nm was obtained in the simulation.

Interference between different diffracted orders of an optical grating can also be used for displacement sensing. With an input displacement of Δd, phase difference is generated between different diffracted beams due to the Doppler effect. Assuming that ±1 th-order diffracted breams are generated from an optical grating with a period of Λ, the phase difference between ±1 th-order beams can be given by [105]

(12) $$\Delta \phi =\frac{4\pi }{\Lambda }\Delta d$$

**Figure 13:**
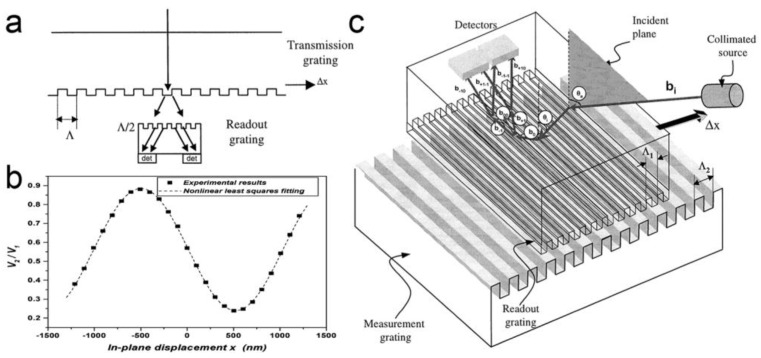
(**a**) Principle of displacement sensing based on diffractive interference [105]. (**b**) Output signal as a function of in-plane displacement [116]. (**c**) Schematic diagram of a miniaturized displacement sensor based on diffractive interference [105].

Optical interference happens when the two beams are recombined by using a second optical grating with a period of Λ/2, which translates the phase difference into a change of intensity (as shown in Figure 13a,b). As shown in Equation (12), the phase difference in the diffractive interference scheme, which is independent of the wavelength of the input light, is only related to the period of the optical gratings and the input displacement. As a result, a better resolution is usually expected with a smaller period of grating. In 2002, Y. Jourlin et al. established a model for diffractive interferometric displacement sensing by using multiple gratings, demonstrating the possibility for a miniaturized displacement sensor based on a reflective structure (as shown in Figure 13c) [105]. Later, they reported a silicon-integrated MOEMS displacement sensor using a standard 0.6 μm CMOS process, achieving a high integration of the encoder [106]. In 2006, G.Y. Zhou et al. reported a multi-freedom-of-degree MOEMS sensor associating the FP resonance with diffractive interference [116]. A resolution of 0.23 nm/Hz^1/2^ and 0.03 nm/Hz^1/2^ for in-plane and out-of-plane displacement sensing were obtained, respectively.

**Figure 14:**
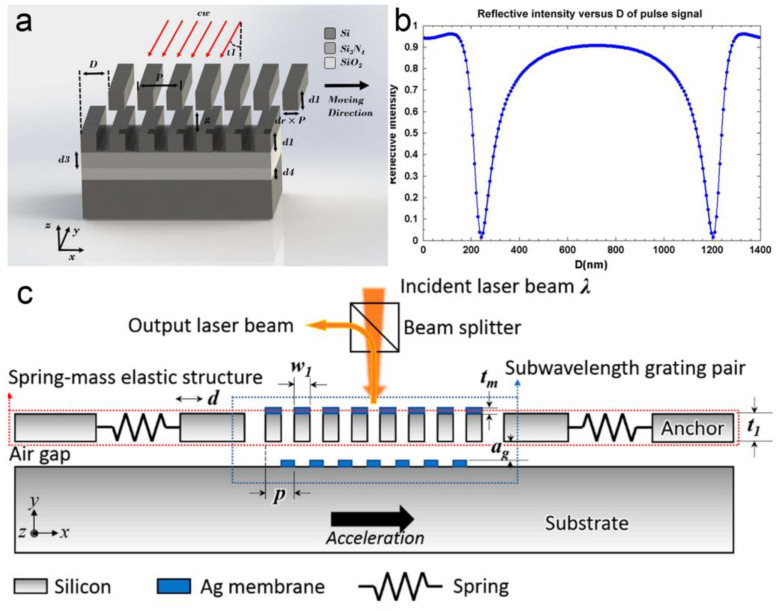
(**a**) Schematic diagram of a reflective two-grating structure [119]. (**b**) Relationship between the reflective intensity and in-plane displacement [119]. (**c**) Schematic diagram of a MEOMS accelerometer based on Wood’s anomalies of diffractive gratings [120].

Another method for displacement sensing is based on Wood’s anomalies of optical gratings. A tremendous increase or decrease in the intensity of certain diffracted orders can be observed due to a small variation in the physical parameters, such as relative locations between two gratings (as shown in Figure 14a,b) [117,118]. As a result, the optical sensitivity can be greatly enhanced to be at 1%/nm level by carefully setting the geometries for a two-grating structure. In 2015, C. Wang et al. designed a MOEMS displacement sensor using anomalous diffraction in a two-grating reflective structure [119]. An optical sensitivity of 2.5%/nm was obtained in the simulation by carefully tuning the air gap between the two gratings. In 2018, Q. Lu et al. designed a MOMES accelerometer (as shown in Figure 14c) [120]. With a decrease of optical reflection over 80% within a small lateral shift of less than 0.15 μm of a movable grating, an optical displacement sensitivity of 1.8%/nm was demonstrated in simulation. In the next year, M. Li et al. designed a micro-gyroscope based on a similar two-gratings structure [121]. The influence of the air gap on the diffraction efficiency was analyzed in theory, showing a sensitivity of 0.22%/nm.

**Figure 15:**
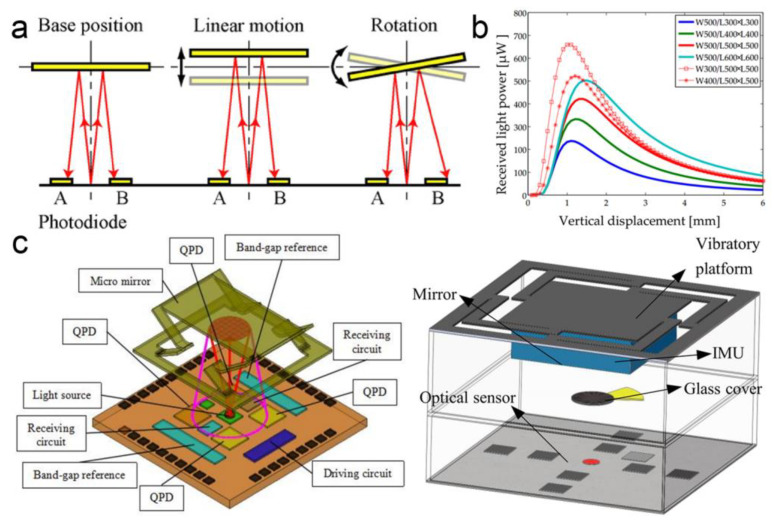
(**a**) Principle of the triangulation measurement [122]. (**b**) Received light power of photodiodes with a vertical displacement of the reflector [124]. (**c**) Schematic diagrams of MOEMS displacement sensors based on triangulation measurement. Structures with and without a covering glass are shown in the left and the right images, respectively [124,127].

For the methods mentioned above, multi-layer structures consisting of optical gratings and metal films are typically required. Using triangulation measurement based on a single reflective layer, many compact structures can be achieved. A divergent light beam is typically used in this case. The beam from an optical source is detected by several detectors located on the different positions of the same substrate after being reflected by a reflector. The intensities of the light reaching different detectors change as the reflector rotates or moves linearly (as shown in Figure 15a,b) [122,123]. For example, as the reflector moves vertically towards the optical detectors, the size of the reflected beam decreases as a reason for a smaller optical path. The outputs of the detectors decrease as well for less overlapping between the reflective beam and the detectors. In this way, I. Ishikawa et al. fabricated an integrated micro-displacement sensor with a measuring range of up to 1.8 mm in 2007 [122]. And a resolution going down to 20 nm was also demonstrated. After that, several works focused on a similar structure based on two-dimensional monolithically integrated photodiodes (as shown in Figure 15c) [35,124,125]. In 2018, Y. Du et al. fabricated a high-dynamic micro-vibrator with an integrated optical displacement detector for multi-axial vibration [126]. The displacement detector was integrated into the top of the package to measure the multi-degree-of-freedom vibration within a range of 500 μm. In 2020, H. Zhan et al. reported an improved design by using a layout optimization and a shading glass cover (as shown in Figure 15c) [127]. The glass cover located behind the vertical-cavity surface-emitting laser was used to absorb interference light and help calibrate the long-term signal drift.

**Figure 16:**
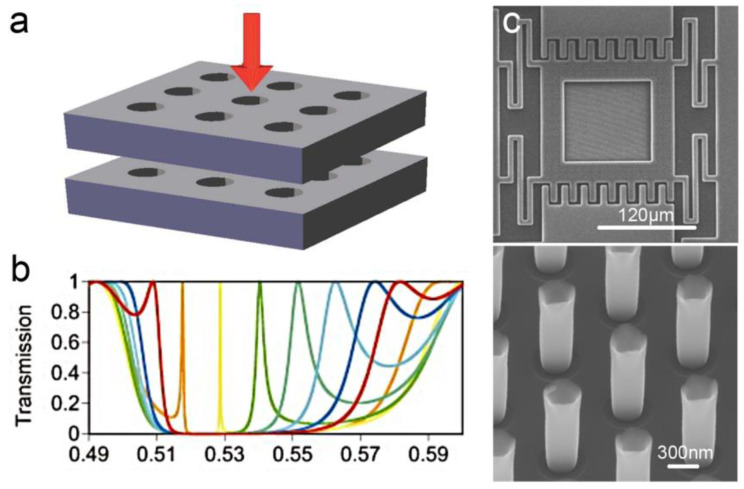
(**a**) Schematic diagram for a two-layer photonic crystal structure [82]. (**b**) Transmission of a two-layer photonic crystal [84]. The colors of the solid lines represent different spacings between the two layers. (**c**) SEM images of a fabricated MOMES displacement sensor based on photonic crystals [22].

Evanescent tunneling between photonic crystals has also been reported for displacement sensing. As two photonic crystal slabs are located close to each other, the guided resonances in the slabs are coupled through an evanescent tunneling process in addition to the free-space propagating (as shown in Figure 16a). As a result, the distance between the two slabs changes the optical reflection. The measuring range, in this case, is usually limited to no more than 1 μm by an evanescent coupling process. In 2003, W. Suh et al. proposed a mechanically tunable photonic crystal structure consisting of coupled photonic crystal slabs, demonstrating a distance-induced change of optical transmission [84]. In 2005, the same group further analyzed the evanescent tunneling between guided resonances in photonic crystal slabs [82]. The transmission properties were analyzed by using a temporal coupled-mode theory. A Fano line shape of the transmission indicated the existence of a guided resonance. The simulated results demonstrated a displacement measuring range of 250 nm. In 2010, S. Hadzialic et al. fabricated a MOEMS displacement sensor using a double-layer reflective structure, showing a reflectivity change of over 80% for a displacement of 115 nm [22]. The sensor was formed from a photonic crystal slab and a silicon substrate with a pillar array on it. The reflectivity changed with a relative displacement between the pillar array and the slab.

## 5. Conclusions and Outlook

This review summarizes the development of MOEMS sensing techniques in past decades. Two different categories are introduced: the guided-wave-based and the free-space-based scheme, respectively. The guided-wave-based approaches are generally used for high-precision measurement within a small range from sub-micrometer to sub-millimeter level. A resolution going down to 0.1–10 fm/Hz^1/2^ level has been demonstrated. In contrast, the free-space-based types generally show a relatively larger measuring range of up to several millimeters with a resolution ranging from sub-pm/Hz^1/2^ to nm/Hz^1/2^ level. The principle, structure and performance for different categories are discussed. By taking advantage of both the MOEMS and optical sensing, these MOMES displacement sensing techniques have been demonstrated as excellent solutions for applications such as photoacoustic spectroscopy, high-precision positioning and automation.

Due to the rapid development of information techniques and industries, future MOMES displacement sensing techniques are facing several challenges and opportunities:

Full integration: Despite the great improvement achieved in past decades, full integration remains a challenge for most MOEMS displacement sensors. In most cases, displacement measurement is operated by using a MOEMS sensing chip associated with discrete components such as optical source, splitter and detector. The full integration of the optical source, sensor and detector is the ultimate goal for MOEMS displacement sensing. Considering the rapid development of heterogeneous integrating techniques associated with optimized structures to simply the interrogation and read-out systems, higher integration is expected.

Higher performance: Although high resolution with a measurement imprecision equal to the standard quantum limit in a tiny range has been reported [42], ultrasensitive displacement sensing with a relatively large measuring rang (e.g., sub-millimeter to millimeter scale) is still in high demand. Techniques such as metamaterial and surface plasmon polariton, which can introduce stronger optical confinement and matter-light interaction, may be used for much higher sensitivity and integration for a long-rang measurement. Data-driven approaches such as artificial neural networks can also be used in the linearization and calibration processes of the sensors for ultrahigh precision.

Intelligence sensing: With the rapid development of information techniques and industries, sensors with higher intelligence are in great demand. Higher functionality for data processing and logic controlling is needed, requiring the integration of more functional modules on a single chip. What is more, considering the big data needed for ultra-sensitive sensing, machine learning may be used to dramatically promote the development of intelligence displacement sensing.
